# Freeze–Thaw‐Induced Physically Cross‐Linked Poly(Vinyl Alcohol)/Alginate Hydrogels Incorporating Silver‐Doped Zinc Oxide and Clove Oil

**DOI:** 10.1002/bip.70098

**Published:** 2026-04-05

**Authors:** Büşra Mutlu

**Affiliations:** ^1^ Bursa Technical University, Central Research Laboratory Bursa Türkiye

**Keywords:** clove oil, hydrogel, poly(vinyl alcohol), silver‐doped zinc oxide, sodium alginate

## Abstract

This study reports the fabrication and comprehensive characterization of multicomponent silver‐doped zinc oxide (Ag/ZnO)–poly(vinyl alcohol)/alginate(PVA/Alg) biocomposite hydrogels enriched with clove oil (CO), prepared via a scalable and solvent‐free freeze–thaw (F–T) process. The F–T treatment generated physically cross‐linked hydrogel networks with preserved structural integrity and favorable mechanical performance. Morphological analysis revealed a homogeneous and interconnected porous architecture at low CO contents, while contact‐angle measurements confirmed hydrophilic surfaces with composition‐dependent wettability. X‐ray diffraction and Fourier transform infrared spectroscopy verified the successful incorporation of Ag/ZnO and CO into the PVA/Alg matrix without disrupting the overall polymer network. Barrier performance was strongly governed by CO content. Increasing CO loading reduced water vapor transmission through the formation of hydrophobic domains, while oxygen permeability reached a maximum at 1 wt.% CO, highlighting tunable mass transport behavior. The hydrogels exhibited rapid swelling, sustained hydration stability for up to 72 h, and a composition‐dependent CO release behavior. Collectively, these findings elucidate clear structure–processing–property relationships in Ag/ZnO‐incorporated PVA/Alg hydrogels containing CO and demonstrate their promise as multifunctional materials for further investigation in wound dressing applications.

## Graphical abstract

Freeze–thaw‐induced physically cross‐linked PVA/alginate hydrogels incorporating Ag/ZnO and clove oil exhibit improved structural integrity, tunable barrier properties, and controlled release behavior, highlighting their potential as multifunctional wound dressing materials.

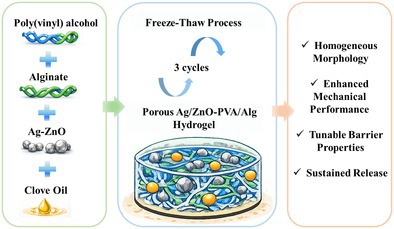

## 1 Introduction

Burn wounds, which arise from exposure to extreme thermal or chemical agents, represent a major global healthcare challenge [1]. Beyond the immediate tissue damage, such injuries are characterized by prolonged inflammatory responses and impaired re‐epithelialization, both of which significantly delay the healing process [1, 2, 3]. Effective burn wound management therefore requires not only protection of the injured site but also the establishment of a local environment that supports tissue regeneration and functional recovery [2, 3, 4].

An effective wound dressing is expected to preserve a hydrated wound interface, shield the injured area from external contaminants, and reduce tissue damage associated with dressing replacement. Traditional dressing materials, including cotton gauze and bandages, frequently fall short of these requirements by excessive absorbtion wound fluids, drying the wound surface and adhering to regenerating tissue, which can result in pain and secondary injury during removal. These limitations have driven the development of next‐generation wound dressings designed to actively support the healing process [5, 6]. Hydrogels have gained increasing attention in wound management owing to their hydrated three‐dimensional polymer networks formed through various cross‐linking approaches [7]. The inherent porosity and hydrophilicity of hydrogels facilitate effective uptake of wound exudate while sustaining a hydrated environment that supports cell movement and tissue regeneration. Moreover, hydrogels exhibit adjustable physicochemical and mechanical characteristics, enabling them to approximate the viscoelastic response of soft tissues and conform to non‐uniform wound surfaces [8, 9, 10]. Hydrogels can be prepared through chemical or physical cross‐linking strategies. Chemical cross‐linking typically relies on reactive agents or initiators, which may introduce cytotoxic residues and limit precise control over network formation. In contrast, physical cross‐linking methods avoid the use of chemical reagents and are therefore particularly attractive for biomedical applications. Among these approaches, repeated freeze–thaw (F–T) cycling has gained considerable attention, as it enables the formation of stable hydrogel networks through crystallite formation and hydrogen‐bonding interactions, while remaining solvent‐free and non‐toxic [11, 12, 13].

Poly(vinyl alcohol) (PVA) is commonly employed as a synthetic matrix for hydrogels fabricated through physical cross‐linking induced by F–T cycling. The presence of uniformly distributed hydroxyl groups along the PVA backbone promotes crystallite formation via interchain hydrogen bonding during F–T treatment, yielding mechanically stable and biocompatible hydrogel networks [14]. Although PVA hydrogels are widely recognized as promising wound dressing materials, single‐component systems may not inherently meet the multifactorial performance requirements of advanced wound care. Therefore, composite PVA‐based hydrogels have been increasingly investigated to achieve integrated multifunctionality [15, 16, 17]. Polymer blending offers an effective route to overcome this limitation by combining complementary properties within a single matrix [11, 14]. In this context, the blending of polymers has emerged as a practical strategy to enhance the clinical performance of hydrogel dressings. Sodium alginate (Alg), a naturally derived anionic polysaccharide composed of mannuronic and guluronic acid units, has been extensively employed in wound care owing to its biocompatibility, hydrophilicity, and high exudate absorption capacity [18, 19, 20, 21]. When exposed to wound exudate, Alg undergoes gelation, forming a soft, conformable layer that adapts to the wound topology and supports a hydrated healing interface. When blended with PVA, Alg contributes to improved water retention, mechanical performance, and thermal stability through synergistic interactions between the polymer chains [22]. Consequently, PVA/Alg blends have attracted growing interest as hydrogel matrices for burn wound dressings that combine the advantages of synthetic and natural polymers [23].

To further extend the functional performance of polymeric hydrogels, the incorporation of inorganic fillers has emerged as an effective materials design strategy. Various nanofillers, including silver‐doped silicon oxide (Ag/SiO_2_) or graphene oxide (GO), and other metal‐based nanoparticles have been incorporated into hydrogel systems to enhance functional performance, mechanical stability, and structural integrity of the polymer network [24, 25, 26]. Among these candidates, ZnO‐based nanostructures have attracted significant attention in biomedical materials due to their reported bioactive functionality and compatibility with polymeric matrices. In this context, silver‐doped zinc oxide (Ag/ZnO) powders represent a multifunctional bioceramic additive that may contribute to mechanical reinforcement, structural stability, and additional biofunctional characteristics. The reinforcing effect of Ag/ZnO within the PVA/Alg matrix is mainly attributed to interfacial interactions between the ceramic particles and the polymer network. Hydroxyl groups of PVA and carboxylate groups of Alg can interact with the ZnO surface through hydrogen bonding and coordination interactions, which may restrict polymer chain mobility and facilitate stress transfer within the composite [27]. In addition, previous studies have shown that the biofunctional behavior of Ag/ZnO systems with the release of Ag^+^ and Zn^2+^ ions, which may influence microbial membranes and related biological responses [28]. These combined effects suggest that Ag/ZnO may contribute to both the structural stability and overall functional performance of polymer‐based hydrogel systems [29, 30]. Incorporating Ag/ZnO into polymer‐based hydrogel matrices provides a means to improve network reinforcement and tune functional material properties relevant to wound care applications [30]. Beyond inorganic fillers, the incorporation of bioactive agents represent an additional strategy to enhance wound‐healing performance. Essential oils derived from aromatic plants have attracted increasing interest due to their reported antioxidant, anti‐inflammatory, and bioactive properties. Nevertheless, the practical use of essential oils is frequently constrained by their high volatility and susceptibility to degradation under environmental conditions such as heat, light, and oxidative stress. Entrapment of essential oils within hydrogel networks represents an effective strategy to enhance their stability and regulate their release over prolonged durations [31, 32, 33]. Among essential oils, clove essential oil (CO), obtained from *Syzygium aromaticum* , is particularly notable due to its high content of eugenol and related phenolic compounds. CO is classified as generally recognized as safe (GRAS) and has been reported to exhibit antioxidant, anti‐inflammatory, and analgesic effects, making it a promising bioactive component for wound‐related material systems. Previous studies have highlighted the relevance of CO‐loaded polymeric systems in wound‐related applications, including systems investigated against common wound‐associated pathogens and infected burn conditions [34, 35, 36].

Despite extensive research on PVA‐ and Alg‐based hydrogels, most previously reported systems rely on single fillers or chemically cross‐linked matrices, and only limited studies have explored the integration of multifunctional inorganic fillers together with natural bioactive agents within physically cross‐linked PVA/Alg networks. The integration of bioceramic fillers into physically cross‐linked PVA/Alg networks fabricated via F–T processing therefore remains largely unexplored at the materials‐design level. In particular, solvent‐free hydrogel systems that concurrently incorporate Ag/ZnO and clove oil within a physically cross‐linked F–T framework have not yet been reported with a systematic structure–processing–property analysis. Although clove oil is incorporated as a hydrophobic liquid component, it is dispersed within the continuous PVA/Alg hydrogel network formed via F–T cross‐linking, and no organic solvent is used during network formation. Accordingly, the developed materials retain the structural characteristics of composite hydrogels rather than oleogel‐type systems. To address this gap, the present study investigates the design and materials‐level performance of Ag/ZnO‐reinforced PVA/Alg hydrogels enriched with clove oil. The combined effects of compositional modulation and F–T processing on network formation, mechanical response, surface wettability, barrier behavior, and mass‐transport properties are comprehensively evaluated. By explicitly correlating processing conditions with tunable physicochemical responses, this work establishes a rational materials‐design framework for multifunctional, physically cross‐linked hybrid hydrogels. In this context, wound dressing is presented as a relevant biomedical application area for further investigation.

## 2 Material and Methods

### 2.1 Materials

Silver‐doped zinc oxide (Ag/ZnO) powders were synthesized using zinc nitrate hexahydrate and silver nitrate (Sigma‐Aldrich, Germany). Poly(vinyl alcohol) (PVA, average Mw ≈44,000 g/mol) and sodium alginate (Alg) were used as polymeric components and obtained from commercial suppliers. Clove essential oil (CO) was sourced from a local producer (Bursa, Türkiye), while Tween 80 was used as a non‐ionic emulsifier. Dulbecco's phosphate buffered saline (PBS, pH 7.4) was obtained from Gibco (USA). All other chemicals, including analytical‐grade salts for simulated body fluid (SBF) preparation, were supplied by Merck (Germany) and used as received. Distilled water was employed throughout all experimental procedures.

### 2.2 Powder Synthesis

Ag‐doped ZnO powders were prepared via a precipitation‐based route in which an aqueous sodium hydroxide solution (1 M) was slowly introduced into a mixed precursor solution containing zinc nitrate hexahydrate (0.1 M) and silver nitrate (0.075 M) under continuous stirring [37]. The reaction system was maintained at 60°C for 2 h, followed by aging for 24 h to promote complete precipitation. The resulting solids were recovered by centrifugation, repeatedly washed with deionized water and ethanol until neutral pH was reached, dried at 80°C for 3 h, calcined at 350°C for 4 h, and gently ground prior to use.

X‐ray diffraction analysis was employed to identify the crystalline phases and assess the crystallite size of the synthesized powders, using a Bruker AXS D8 diffractometer equipped with Cu–Kα radiation (λ = 1.5406 Å) and operated at 45 kV and 40 mA. Diffraction patterns were recorded within a 2*θ* interval of 25°–100° and interpreted with reference to the ICDD database. Crystallite size values were calculated from diffraction peak broadening using the Scherrer method. Particle size distribution was analyzed by laser diffraction (Malvern Mastersizer 3000E). In addition, microstructural features and elemental composition were investigated by field‐emission scanning electron microscopy combined with energy‐dispersive spectroscopy (FE‐SEM/EDS, Zeiss Gemini 300). The specific surface area of the powders was determined via Brunauer–Emmett–Teller (BET) analysis using an Anton Paar NOVA 800 instrument after degassing the samples at 120°C for 12 h.

### 2.3 Preparation of Essential Oil Emulsion

A stable oil‐in‐water emulsion was prepared by combining clove essential oil (10 wt.%), Tween 80 (10 wt.%), and deionized water (80 wt.%), followed by vortex mixing at 3000 rpm for 3 min and ultrasonication for 3 min to ensure uniform dispersion [38]. The chemical composition of clove oil was analyzed by gas chromatography–mass spectrometry (GC–MS, Agilent 7890B) after dilution in n‐hexane, using a programmed oven temperature from 60°C to 250°C at a heating rate of 3°C min^−1^, helium as the carrier gas, and electron impact ionization at 70 eV. Compound identification was achieved through comparison with reference spectra.

### 2.4 Fabrication of Hydrogel Dressings

PVA/Alg‐based hydrogels were prepared by repeated F–T cycling with slight modifications to previously reported procedures [39, 40]. Aqueous PVA (10 wt.%) and Alg (3 wt.%) solutions were prepared separately and mixed at a volume ratio of 1:1 under continuous stirring to obtain a homogeneous polymer blend. For composite formulations, Ag/ZnO powders were incorporated at concentrations of 0.5, 1, and 2 wt.% relative to the total polymer content. Aliquots (20 mL) of the resulting mixtures were poured into Petri dishes and frozen at −20°C for 18 h, followed by thawing at either 4°C or 24°C for 4 h. This F–T cycle was repeated three times to induce physical cross‐linking of the polymer network.

Based on the mechanical performance, hydrogels containing 1 wt.% Ag/ZnO and thawed at 24°C were selected for clove oil incorporation. The prepared emulsion was added to obtain final CO contents of 1, 3, and 5 wt.%, and the hydrogels were designated according to their composition. The resulting hydrogels were designated as PVA/Alg/Z1, PVA/Alg/Z1^1CO^, PVA/Alg/Z1^3CO^, and PVA/Alg/Z1^5CO^ according to their composition. Detailed formulations are summarized in Table 1.

**TABLE 1:** Mixture compositions used for hydrogel preparation.

Sample code	F–T temperature (°C)	Powder ratio (wt.%)	Essential oil ratio (wt.%)
PVA/Alg	4	—	—
PVA/Alg/Z0.5	4	0.5	—
PVA/Alg/Z1	4	1	—
PVA/Alg/Z2	4	2	—
PVA/Alg	24	—	—
PVA/Alg/Z0.5	24	0.5	—
PVA/Alg/Z1	24	1	—
PVA/Alg/Z2	24	2	—
PVA/Alg/Z1^1CO^	24	1	1
PVA/Alg/Z1^3CO^	24	1	3
PVA/Alg/Z1^5CO^	24	1	5

### 2.5 Powder Characterization

The crystalline structure of the synthesized powders was analyzed by XRD analysis using a Bruker AXS D8 diffractometer operated under Cu–Kα radiation. Diffraction patterns were collected within a 2*θ* range of 20°–60° and interpreted using with standard reference data from the ICDD database to determine the existing crystalline phases [37, 41]. Crystallite size was estimated from diffraction peak broadening using the Scherrer equation as given in Equation (1).

(1) $$\;D=\frac{\mathrm{k\lambda}}{\text{βcosθ}}$$

In this equation, *D* denotes the average crystallite size, *k* is the Scherrer constant (0.9), *λ* represents the wavelength of the incident X‐ray radiation (1.5406 Å), *β* corresponds to the full width at half maximum (FWHM) of the diffraction peaks, and *θ* is the Bragg diffraction angle.

Particle size distribution of the Ag/ZnO powders was analyzed by laser diffraction using a Malvern Mastersizer 3000E system. The microstructural features and elemental composition were examined by field‐emission scanning electron microscopy coupled with energy‐dispersive spectroscopy (FE‐SEM/EDS, Carl Zeiss Gemini 300), operated at an accelerating voltage of 10 kV. Prior to imaging, the powder samples were coated with a thin Au–Pd layer to enhance surface conductivity.

Functional group analysis was carried out using Fourier‐transform infrared (FTIR) spectroscopy (Thermo Nicolet iS50) over the spectral range of 650–4000 cm^−1^ with a resolution of 4 cm^−1^ and an average of 32 scans. Specific surface area measurements were performed via Brunauer–Emmett–Teller (BET) analysis using an Anton Paar NOVA 800 analyzer. Before BET measurements, samples were degassed at 120°C for 12 h to remove adsorbed moisture and gases.

### 2.6 Gas Chromatography–Mass Spectrometry (GC–MS) Examination of Essential Oil

The chemical composition of clove essential oil was analyzed using gas chromatography–mass spectrometry (GC–MS, Agilent 7890B). Prior to analysis, 20 μL of CO was diluted in 1.5 mL of n‐hexane, and 0.2 μL of the solution was injected into the system. The oven temperature was programmed from 60°C to 250°C at a heating rate of 3°C min^−1^ and held for 5 min. Helium was used as the carrier gas with a split ratio of 1:5. Mass spectra were recorded at an ionization energy of 70 eV over an m/z range of 30–550, and compound identification was performed by comparison with reference standards.

### 2.7 Hydrogel Dressing Characterization

#### 2.7.1 Physical Crosslinking of Ag/ZnO‐(PVA/Alg)‐Based Hydrogels

The efficiency of physical cross‐linking induced by F–T cycling was evaluated through visual inspection and gel fraction measurements. Gel fraction analysis was employed to quantify the extent of insoluble network formation after repeated F–T cycles, which reflects the degree of crystallite‐mediated cross‐linking within the hydrogel structure. Hydrogel samples were cut into square specimens (1.5 cm × 1.5 cm) and dried in an oven at 50°C for 6 h to obtain the initial dry weight ($W_{0}$). The dried samples were then immersed in distilled water for 24 h to remove soluble polymer fractions until equilibrium swelling was achieved. Subsequently, the remaining gels were dried again at 50°C and weighed (*W*_*e*_). The gel fraction was calculated as the ratio of the final dry weight to the initial dry weight, according to Equation (2) [40, 42]:

(2) $$\;\text{Gel}\;\text{fraction}\;\left(\%\right)=\frac{W_{e}}{W_{0}}\times 100$$

#### 2.7.2 Mechanical Characterization of Ag/ZnO‐(PVA/Alg)‐Based Hydrogels

The mechanical response of the hydrogel formulations was assessed by uniaxial tensile testing using a universal testing system (Testform/AS1) fitted with a 1 kN load cell. Rectangular specimens with dimensions of 5 cm × 1 cm and an average thickness of approximately 0.2 mm were subjected to tensile deformation at room temperature under a constant crosshead speed of 5 mm min^−1^ until rupture. The mechanical tests were performed on dried hydrogel films obtained after freeze–thaw processing to ensure dimensional stability and reproducible measurements. The resulting stress–strain curves were analyzed to determine the tensile strength and elongation at break. For each formulation, five independent specimens were tested, and the results are presented as mean ± standard deviation.

#### 2.7.3 Morphological and Surface Characterization of Essential Oil‐Ag/ZnO‐(PVA/Alg)‐Based Hydrogels

Surface and crosssectional morphologies of the CO–loaded Ag/ZnO–PVA/Alg hydrogels were examined by field‐emission scanning electron microscopy (FE‐SEM, Zeiss Gemini 300). To expose the internal microstructure, hydrogel samples were cryo‐fractured in liquid nitrogen prior to imaging. All specimens were subsequently coated with a thin Au–Pd layer to enhance electrical conductivity during observation.

Surface wettability was evaluated through static contact angle measurements using the sessile drop technique. Deionized water and artificial sweat solution (ASS) were employed as probe liquids, and droplets with a volume of 5 μL were carefully deposited onto the hydrogel surface. A small droplet volume was used to minimize liquid absorption by the hydrogel matrix. Images were recorded after 5 s of contact time. The contact angle was recorded immediately after droplet deposition in order to minimize possible artifacts associated with droplet imbibition. Measurements were conducted at 21°C and 50% relative humidity, and the reported contact‐angle values represent the average of three independent measurements.

#### 2.7.4 Structural and Thermal Characterization of Essential Oil‐Ag/ZnO‐(PVA/Alg)‐Based Hydrogels

The crystalline structure of the hydrogel samples was analyzed by X‐ray diffraction (XRD, Bruker AXS D8) using Cu–Kα radiation operated at 40 kV and 40 mA. Diffraction patterns were collected over a 2*θ* range of 15°–50° with a step size of 0.02°, and phase identification was performed using the ICDD database.

Fourier transform infrared (FTIR) spectroscopy (Thermo Nicolet iS50) was employed to investigate functional groups and possible intermolecular interactions between polymers, Ag/ZnO powders, and clove oil. Spectra were recorded in the range of 650–4000 cm^−1^ with a resolution of 4 cm^−1^, averaging 32 scans per sample.

Thermal behavior was evaluated using differential scanning calorimetry (DSC, TA Instruments DSC25). Approximately 7–8 mg of each sample was sealed in aluminum pans and heated from 10°C to 350°C at a rate of 10°C/min under a nitrogen atmosphere. Melting transitions were determined from the peak maxima of the first heating cycle.

Thermal stability was further assessed by thermogravimetric analysis (TGA, TA Instruments SDT 650). Samples were heated from 20°C to 400°C at the same heating rate under a nitrogen flow of 10 mL/min, and weight‐loss profiles were recorded.

#### 2.7.5 Barrier Characterization of Essential Oil‐Ag/ZnO‐(PVA/Alg)‐Based Hydrogels

Water vapor transmission rate (WVTR) of the hydrogel samples was determined using a gravimetric cup method based on ASTM E96 with minor modifications. Hydrogel samples were sealed over poly(methyl methacrylate) cups containing anhydrous desiccant (0% RH), and the assemblies were stored at ambient temperature and 50% RH. Weight changes were monitored at 2 h intervals over 48 h using an analytical balance. The WVTR of the sample was then calculated using Equation (3):

(3) $$\text{WVTR}=\left(\frac{w}{t}\right)\times \frac{1}{A}$$

where w is the weight change (g), t is the time (h), and A is the exposed sample area (m^2^).

Oxygen transmission rate (OTR) measurements were performed according to ASTM D1434–23 using a Labthink VAC‐V2 gas permeation analyzer. Measurements were conducted at 23°C and 0% RH using oxygen as the test gas. Sample thickness was measured using a digital micrometer Mitutoyo (293–821, resolution: 0.001 mm) at five random positions per sample (*n* = 3 independent specimens) and reported as mean ± SD.

#### 2.7.6 In Vitro Biological Characterization of Essential Oil‐Ag/ZnO‐(PVA/Alg)‐Based Hydrogels

The wound exudate absorbency experiments were conducted in simulated wound fluid (SWF), prepared according to the procedure described in a previous report [37]. Briefly, 0.68 g of sodium chloride (NaCl), 0.22 g of potassium chloride (KCl), 2.5 g of sodium hydrogen carbonate (NaHCO_3_), and 0.35 g of sodium dihydrogen phosphate (NaH_2_PO_4_) were dissolved in 100 mL distilled water. Then, the pH of SWF was adjusted to 8 ± 0.2. In the experiment, a sponge cut to fit a glass Petri dish was completely wetted with SWF. The pre‐weighed hydrogel dressing samples were placed on the upper surface of the SWF‐wetted sponge. Thus, only one surface of the samples was in contact with the wet sponge. The soaked samples were weighed at different times until an equilibrium was achieved, and the wound exudate uptake of the samples was determined using the following Equation (4):

(4) $$\mathrm{SWF}\;\text{uptake}\;\left(\%\right)=\frac{W_{t}-W_{0}}{W_{0}}\times 100$$

*W*_*t*_ and *W*_o_ are the weight after uptake and initial weight of the samples, respectively.

The in vitro release behavior of CO from the hydrogels was investigated in PBS (pH 7.4) at 37°C to simulate physiological dermal conditions. CO‐loaded hydrogel samples weighing 10 mg were immersed in 10 mL of PBS under static conditions, and aliquots were withdrawn at predetermined time points [43]. The withdrawn medium was replaced with fresh PBS to maintain sink conditions. Released CO was quantified by UV–visible spectroscopy at 280 nm, corresponding to the characteristic absorption band of eugenol, the major phenolic component of CO. A calibration curve was established using CO emulsions of known concentrations prepared under identical experimental conditions. Tween 80 was used as a nonionic surfactant to facilitate the dispersion of hydrophobic CO in the aqueous PBS medium during spectroscopic measurements. To minimize potential spectral interference, blank solutions containing PBS and Tween 80 without CO were measured and used for baseline correction. In addition, control hydrogels without CO were analyzed to verify that PVA/Alg components did not significantly contribute to the absorbance at 280 nm. All measurements were performed in triplicate to ensure reproducibility and statistical reliability.

Kinetic analysis was conducted by fitting the release data to the Higuchi and Korsmeyer–Peppas models in order to identify the governing transport mechanism of CO release from the polymeric network [44, 45]. The Higuchi model (Equation 5) was employed to evaluate diffusion‐controlled release behavior:

(5) $$Q_{t}=k_{H}t^{1/2}$$

where $Q_{t}$ is the cumulative amount of CO released at time t and $k_{H}$ is the Higuchi release constant.

The Korsmeyer–Peppas model (Equation 6) was used to determine the release exponent (*n*):

(6) $$\;\frac{M_{t}}{M_{\infty }}=Kt^{n}$$

where $\frac{M_{t}}{M_{\infty }}$ is the fractional release at time t, $K_{K}$ is the kinetic constant, and n is the release exponent. Release data were fitted to the Higuchi and Korsmeyer–Peppas models, and the goodness of fit was evaluated using the coefficient of determination (*R* ^2^).

## 3 Results and Discussion

### 3.1 Powder Characterization

**FIGURE 1:**
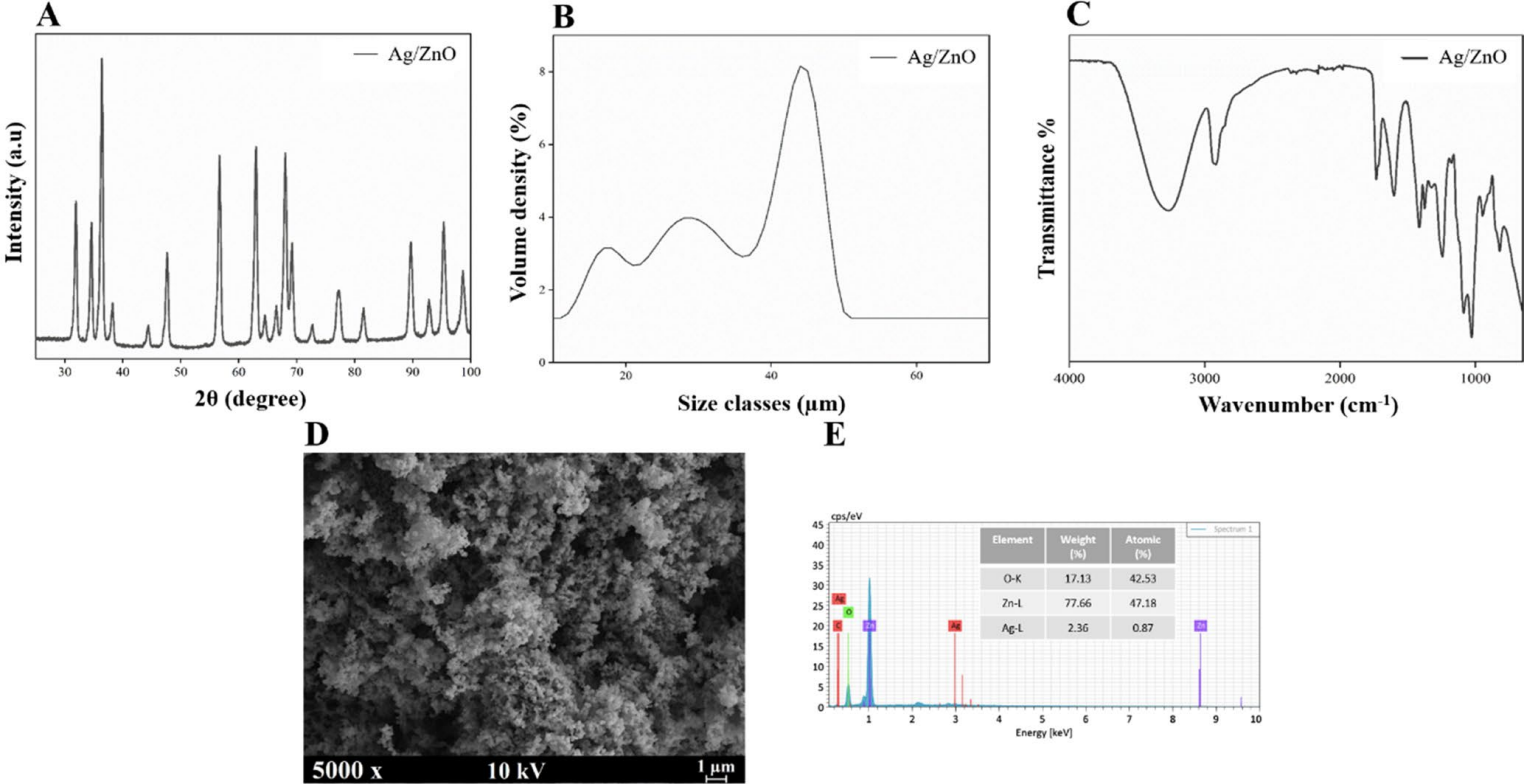
Characterization of the powders: (A) XRD patterns, (B) Mean particle size, (C) FTIR spectra, (D) SEM images, and (E) EDS spectra.

Figure 1A presents the XRD patterns of the synthesized Ag‐doped ZnO powders. The diffraction profiles confirm the formation of well‐crystallized phases, with no detectable secondary reaction products other than ZnO and metallic Ag. The prominent diffraction peaks observed at 2*θ* values of approximately 31.75°, 34.41°, 36.23°, 47.52°, 56.56°, 62.84°, 67.91°, and 69.04° can be indexed to the (100), (002), (101), (102), (110), (103), (112), and (201) crystallographic planes of hexagonal ZnO, respectively (PDF card no. 04‐027‐6897) [46]. These reflections are characteristic of the wurtzite ZnO lattice and indicate that the crystalline structure of ZnO is preserved after Ag incorporation. In addition to the ZnO reflections, weak diffraction peaks corresponding to the face‐centered cubic (FCC) phase of Ag were detected, confirming the successful introduction of silver into the system. Considering the larger ionic radius of Ag^+^ (1.22 Å) compared to Zn^2+^ (0.74 Å), the presence of Ag‐related peaks suggests preferential occupation of interstitial or segregated regions rather than substitutional incorporation into the ZnO lattice, as commonly reported for Ag‐doped ZnO systems [46]. The average crystallite size of the synthesized Ag/ZnO powders was calculated as 17.76 nm.

Particle size analysis based on laser diffraction revealed a average particle size of 34.0 μm (Figure 1B). The discrepancy between the crystallite size and the particle size indicates the formation of micron‐sized agglomerates composed of nanoscale crystallites, which is typical for oxide powders synthesized through aqueous precipitation methods.

Figure 1C presents the FTIR spectra obtained for the synthesized Ag/ZnO powders. A broad absorption feature spanning the 3200–3600 cm^−1^ region is assigned to O—H stretching vibrations arising from surface‐adsorbed water molecules and hydroxyl groups [47, 48]. Bands observed near 2920 and 2850 cm^−1^ are associated with C—H stretching modes originating from residual organic species. The bands observed near 1630 and 1380 cm^−1^ are associated with asymmetric and symmetric stretching modes of acetate groups, while the peak around 1545 cm^−1^ is assigned to C—O stretching vibrations [47, 48]. In addition, absorption bands at approximately 1449 and 1382 cm^−1^ are indicative of carbonate species, which may arise from the formation of Ag_2_CO_3_ during synthesis [34]. A weak band detected near 890 cm^−1^ is attributed to out‐of‐plane bending vibrations of C—H groups associated with alkene‐type structures [35].

The surface morphology of the Ag/ZnO powders, illustrated in Figure 1D, reveals relatively uniform and near‐spherical particles distributed throughout the sample. The absence of pronounced morphological irregularities suggests a homogeneous precipitation process, which is favorable for achieving uniform dispersion of the powders within polymeric hydrogel matrices. Energy‐dispersive spectroscopy (EDS) analysis (Figure 1E) further confirmed the presence of Zn, O, and Ag elements, supporting the compositional integrity of the synthesized powders [26].

The specific surface area of the Ag‐doped ZnO powders was determined to be 7.99 m^2^/g by BET analysis. Such a moderate surface area is advantageous for biomedical composite systems, as it promotes effective interfacial interactions with polymer chains while limiting excessive agglomeration [49]. In the context of hydrogel‐based wound dressings, this balance is expected to facilitate uniform particle dispersion and contribute to mechanical reinforcement without adversely affecting biocompatibility.

### 3.2 Chemical Composition of Essential Oil

**FIGURE 2:**
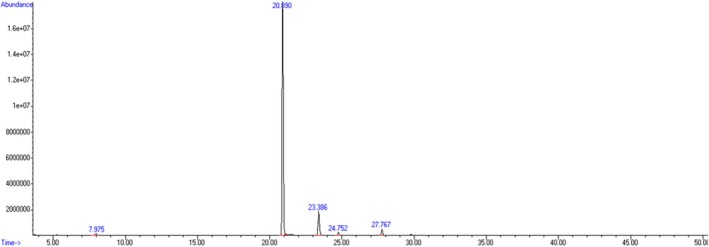
GC–MS chromatogram of CO.

In order to determine the components of CO, each component separated by gas chromatography was subsequently ionized, and mass spectra were recorded. The results are presented in Table 2 and Figure 2. The obtained results were verified by comparing them with the RT (retention time) values of the standard substances in gas chromatography. In the integration results of the chromatograms of CO obtained by distillation from the *Syzygium aromaticum* (L.) buds, the main chemical components were eugenol (86.09%) and caryophyllene (9.89%), which are consistent with previous studies [50, 51, 52].

**TABLE 2:** Relative percentages of the main volatile components identified in CO.

No	RT (min.)	Composition (%)	Compound name
1	7.97	0.69	Eucalyptol
2	20.88	86.09	Eugenol
3	23.38	9.89	Caryophyllene
4	24.75	1.13	Humulene
5	27.76	2.20	Aceteugenol

Abbreviation: RT, retention time.

### 3.3 Hydrogel Characterization

#### 3.3.1 Physical Crosslinking of Ag/ZnO‐(PVA/Alg) Hydrogels

Cross‐linking in hydrogel systems can generally be categorized as either chemical or physical, depending on the nature of interactions formed between macromolecular chains. While chemical cross‐linking is widely employed to fabricate functional hydrogels, the use of reactive cross‐linking agents may lead to unfavorable interactions with bioactive components embedded in the polymer matrix [53]. For this reason, repeated F–T cycles were adopted as a physical cross‐linking strategy to stabilize polymer chains while avoiding potential toxicity associated with chemical cross‐linkers [54, 55].

**FIGURE 3:**
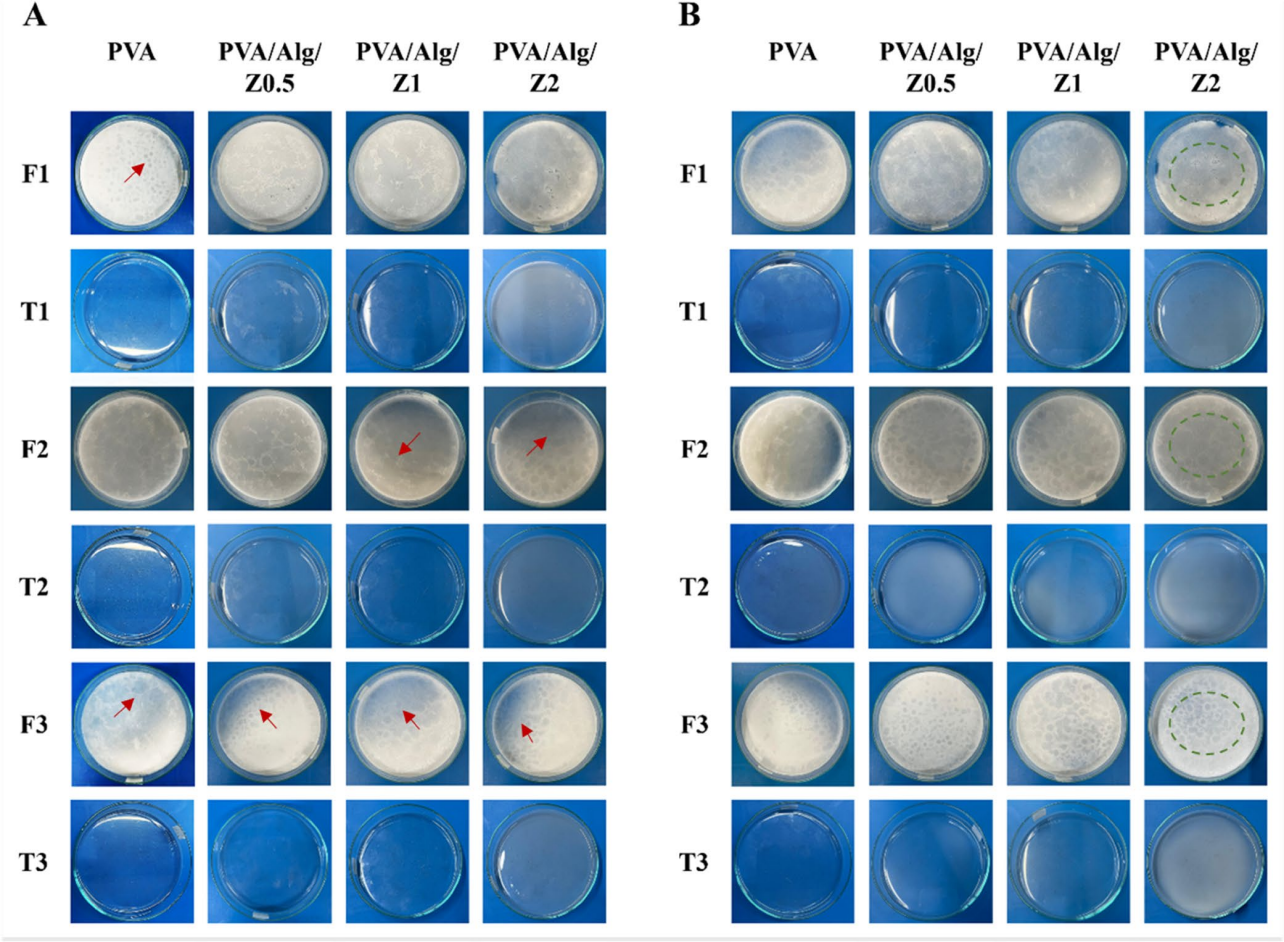
Macroscopic appearances of hydrogels after each freeze‐thaw cycle, respectively, (A) freezing at −18°C and thawing at 4°C and (B) freezing at −18°C and thawing at 24°C. (Red arrows indicate heterogeneous white domains observed in hydrogels thawed at 4°C, whereas green dashed circles highlight the relatively uniform surface of hydrogels thawed at 24°C).

The macroscopic appearance of hydrogels containing different Ag/ZnO contents after F–T processing at 4°C and 24°C are presented in Figure 3. As shown in Figure 3, noticeable differences in the visual appearance of the gels formed after three F–T cycles can be observed. Hydrogels thawed at 4°C exhibited heterogeneous surface features characterized by visible white domains, whereas samples thawed at 24°C showed comparatively more uniform surfaces. The formation of these surface patterns at lower thawing temperatures is attributed to restricted polymer chain mobility, which limits chain rearrangement and promotes the development of crystallite‐rich regions. At a thawing temperature of 24°C, phase separation occurred predominantly between a PVA‐rich phase and a water‐rich phase, indicating more efficient polymer reorganization.

**FIGURE 4:**
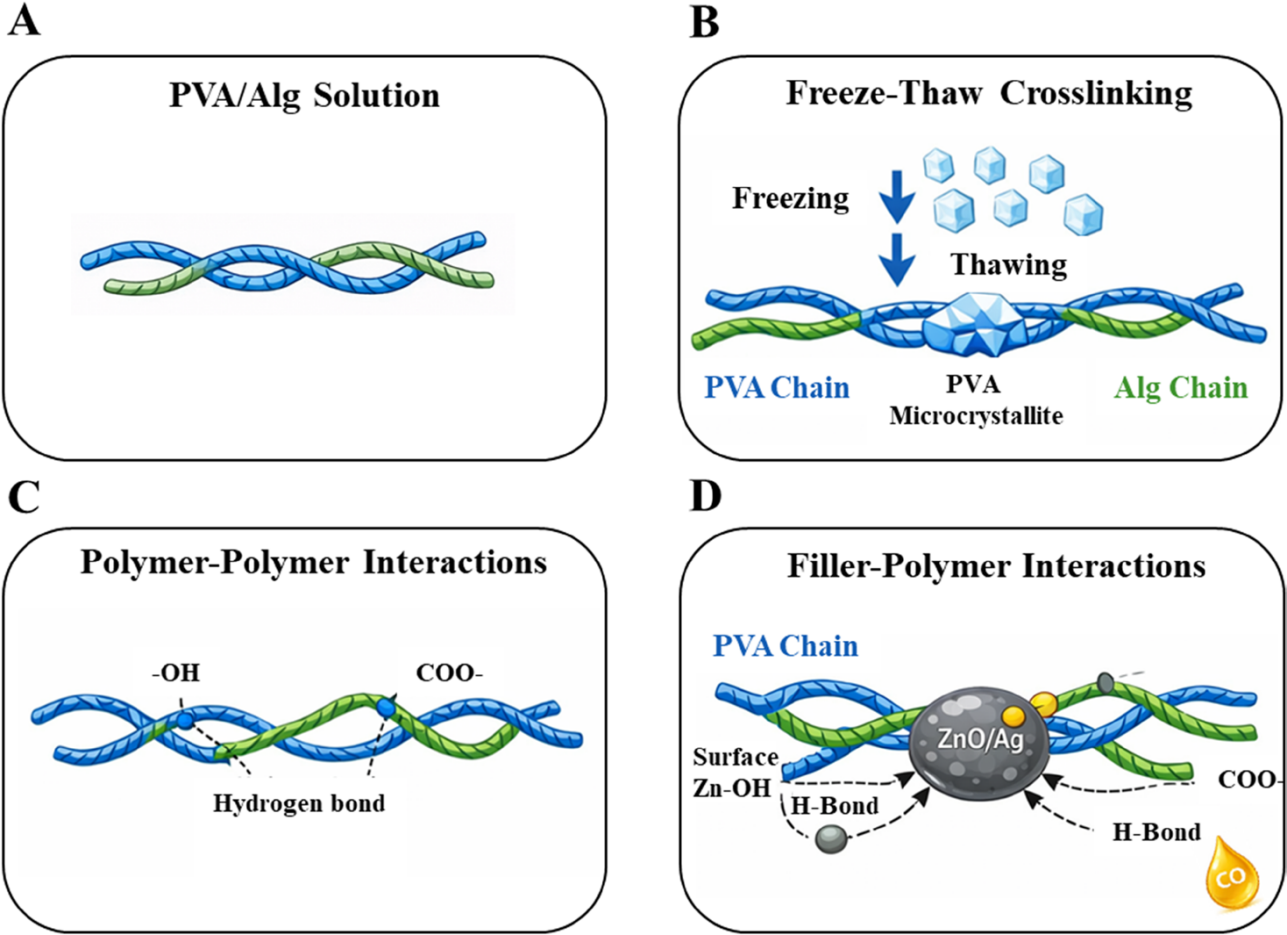
Schematic representation of the interaction mechanisms during freeze–thaw processing of Ag/ZnO‐PVA/Alg‐based hydrogels.

Polymer chains that did not participate in crystallite formation during the early F–T cycles remained partially dissolved and were not fully integrated into the physical gel network. With increasing numbers of F–T cycles, the extent of polymer dissolution decreased, reflecting progressive network stabilization. In all hydrogels, the crystallite area increased as the number of F–T cycles increased, resulting in PVA‐rich phase separation. The increase in the number of F–T cycles highlights the promoting effect of F‐T processing on the microcrystallization of PVA, which is reflected in the properties of the resulting hydrogels [56]. The proposed interaction mechanisms responsible for the formation and reinforcement of the Ag/ZnO–PVA/Alg hydrogel network during F–T processing are schematically illustrated in Figure 4.

**FIGURE 5:**
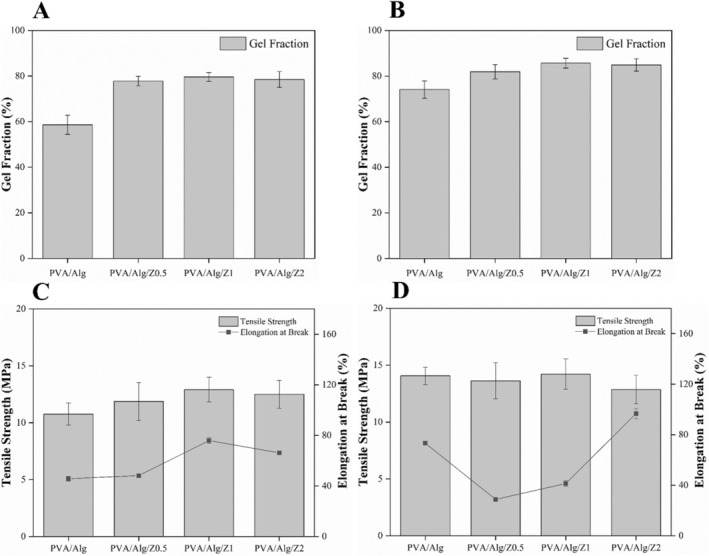
Gel fraction and mechanical properties of hydrogels after each freezing and thawing cycle: (A) Gel fraction of hydrogels freezing at −18°C and thawing at 4°C and (B) freezing at −18°C and thawing at 24°C, Tensile strength and elongation at break of hydrogels after each freezing and thawing cycle, (C) freezing at −18°C and thawing at 4°C and, (D) freezing at −18°C and thawing at 24°C.

Naturally hydrophilic polymers are physically cross‐linked through different F–T cycles to form a three‐dimensional (3D) network. The gel fraction (GF) test, which is used to evaluate cross‐linking efficiency depends on the crystallinity of the polymer network and the degree of cross‐linking. The gelation percentage determined by GF is calculated to assess the influence of processing temperature parameters of water affect it. Moreover, the gelation values of burn wounds treated in cold environments are critical for maintaning structural integrity the wound and causing further deterioration [57]. The gelation percentages of PVA/Alg‐based hydrogel dressings depending on the Ag/ZnO content are presented in Figure 5A and Figure 5B for samples subjected to freezing at −18°C and thawing at 4°C and 24°C, respectively.

The gelation percentage of the PVA sample thawed at 4°C was found to be 58.6% ± 4.15%, while the thawed sample was found to be 74.1% ± 3.75% at 24°C. This increase can be attributed to enhanced crosslinking of PVA and Alg occurred to form a 3D network [42]. However, the lower gelation percentage of the PVA/Alg sample at 4°C revealed that the hydrogel could not crystallize sufficiently at this point and that crystallization could be higher with increasing temperature. On the other hand, the inclusion of Ag/ZnO powders in the structure revealed that the gelation percentage was 77.8 ± 2.1, 79.6 ± 1.96 and 78.5 ± 3.44 for Ag/ZnO0.5, Ag/ZnO1, and Ag/ZnO2 samples thawed at 4°C, while it was found to be 81.9 ± 3.17, 85.7 ± 2.16, and 84.9 ± 2.74 for samples thawed at 24°C. F–T cycles during the preparation of neat PVA/Alg and Ag/ZnO‐doped PVA/Alg‐based hydrogel wound dressings led to the formation of a water‐insoluble polymer network. The obtained gelation percentages not only confirmed the increased gelation percentage of the hydrogels thawed at higher temperatures but also revealed an increased gelation percentage as the amount of Ag/ZnO doping increased. This suggests that the presence of Ag/ZnO powders in the 3D network of the hydrogels could increase the amount of cross‐linking in the hydrogel structure, thus resulting in a greater cross‐link density. This phenomenon, derived from the existence of a strong interaction between the polymer matrix and the powders doped into the structure, has also been reported for polymer composite hydrogels with a wide range of additives and has contributed to improved mechanical properties [58].

As a conclusion, the F–T approach can be effectively used at different temperatures to control the gel fraction in the preparation of biocomposite hydrogels.

#### 3.3.2 Effect of the Ag‐ZnO Powders on the Mechanical Properties of PVA/Alg) Hydrogels

An ideal wound dressing should possess appropriate mechanical properties to minimize damage to regenerating tissue during replacement. Additionally, it should provide sufficient mechanical support to facilitate cell proliferation and tissue remodeling, while maintaining an optimal balance between flexibility and stiffness [2, 59].

Figure 5C,D presenting the stress–strain curves show the mechanical properties of the hydrogels after freezing at −18°C and thawing at 4°C and freezing at −18°C and thawing at 24°C, respectively. The tensile stress corresponds to the maximum stress before failure, while elongation at break indicates the strain at the failure point [60]. The mechanical measurements were conducted on dried samples obtained after F–T processing. Testing under dry conditions allows evaluation of the intrinsic mechanical strength of the polymer network formed during F–T crystallization while minimizing additional plasticization effects caused by absorbed water.

The ultimate tensile strength values for PVA/Alg, PVA/Alg/Z0.5, PVA/Alg/Z1, and PVA/Alg/Z2 which were frozen at −18°C and thawed at 4°C were 10.76 ± 0.98, 11.87 ± 1.67, 12.9 ± 1.09, 12.50 ± 1.23 MPa; the respective elongation at break values were 45.76% ± 1.93%, 48.21% ± 1.02%, 45.88% ± 2.34%, 66.21% ± 1.43%, respectively. When the thawing temperature of hydrogels frozen at −18°C was increased to 24 C, the tensile strength values of PVA/Alg, PVA/Alg/Z0.5, PVA/Alg/Z1, and PVA/Alg/Z2 were measured as 14.06 ± 0.78, 13.63 ± 1.58, 14.21 ± 1.33, and 12.86 ± 1.24 MPa, respectively. The corresponding elongation at break values were 73.22% ± 1.13%, 48.80% ± 1.37%, 41.39% ± 2.43%, and 96.73% ± 3.97%, respectively. The obtained data indicate a positive correlation between thawing temperature and both tensile strength and elongation at break. For instance, increasing the F–T temperature from 4°C to 24 C resulted in a tensile strength of 14.21 MPa and an elongation at break of 41.39% in the PVA/Alg hydrogel doped with 1 wt.% Ag/ZnO powders. These findings demonstrate that modulating the F–T temperature can significantly enhance the mechanical properties of blended hydrogels. This characteristic is particularly valuable for wound dressing applications, as optimizing the F–T temperature allows for improved tensile strength and flexibility, thereby enhancing the hydrogel's ability to conform to complex body contours.

On the other hand, the introduction of Ag/ZnO led to an increase in the ultimate tensile strength of the hydrogel samples. Similarly, in a study investigating the effects of varying concentrations of nanocrystalline silver (Ag) powder on the properties of composite hydrogels, Ag was found to promote the crystallization of PVA, thereby enhancing the mechanical properties of the hydrogel [61]. However, while an initial increase in bioceramic powder content led to improved tensile strength, further increases resulted in a decline due to nanoparticle agglomeration, which impeded the formation of a well‐structured hydrogel network. Comparable findings were reported in another study, where the mechanical performance of wound dressings was enhanced by incorporating Alg into PVA‐based hydrogels in the presence of silver [62]. Moreover, considering the tensile strength of human skin falls within the range of 0.8 to 18 MPa, the findings demonstrate that all fabricated hydrogel dressings possess adequate mechanical strength, making them suitable candidates for wound healing applications [59, 63].

In addition, hydrogel wound dressings reported in the literature generally exhibit relatively limited mechanical strength due to their high water content and loosely cross‐linked polymer networks. For instance, many hydrogel‐based wound dressings show tensile strengths in the range of approximately 0.02–0.3 MPa, depending on polymer composition, crosslinking density, and hydration state [64, 65, 66]. Compared with these conventional hydrogel systems, the PVA/Alg composite hydrogels developed in the present study exhibit markedly higher tensile strength. This improvement can be attributed to the formation of PVA microcrystalline domains during the F–T process, which act as physical cross‐linking junctions within the polymer network [66]. These crystalline regions reinforce the hydrogel matrix and restrict polymer chain mobility, thereby significantly improving the mechanical robustness of the material while maintaining sufficient flexibility for wound dressing applications.

Additionally, the mechanical requirements of wound dressings may vary depending on the anatomical location of application [67]. For instance, in areas with high mobility such as the elbows and knees affected by burn injuries, dressings with enhanced stretchability are preferred, whereas in other regions, increased stiffness may be more suitable. This study demonstrated that the mechanical properties of hydrogel wound dressings can be tailored according to their intended application site by adjusting the ratio of Ag/ZnO incorporated into the formulation. Consequently, in addition to the gel fraction data, the tensile strength of PVA/Alg‐based hydrogels increased with the incorporation of 1 wt.% Ag/ZnO. To further evaluate the additional effects, essential oil was subsequently introduced into the PVA/Alg matrix with varying concentrations, which had been subjected to a F–T process at −18 C and 24 C, respectively. Further analyses were conducted on the resulting formulations: PVA/Alg/Z1, PVA/Alg/Z1^1CO^, PVA/Alg/Z1^3CO^, PVA/Alg/Z1^5CO^.

#### 3.3.3 Morphological Properties of Essential Oil‐Ag/ZnO‐(PVA/Alg) Hydrogels

**FIGURE 6:**
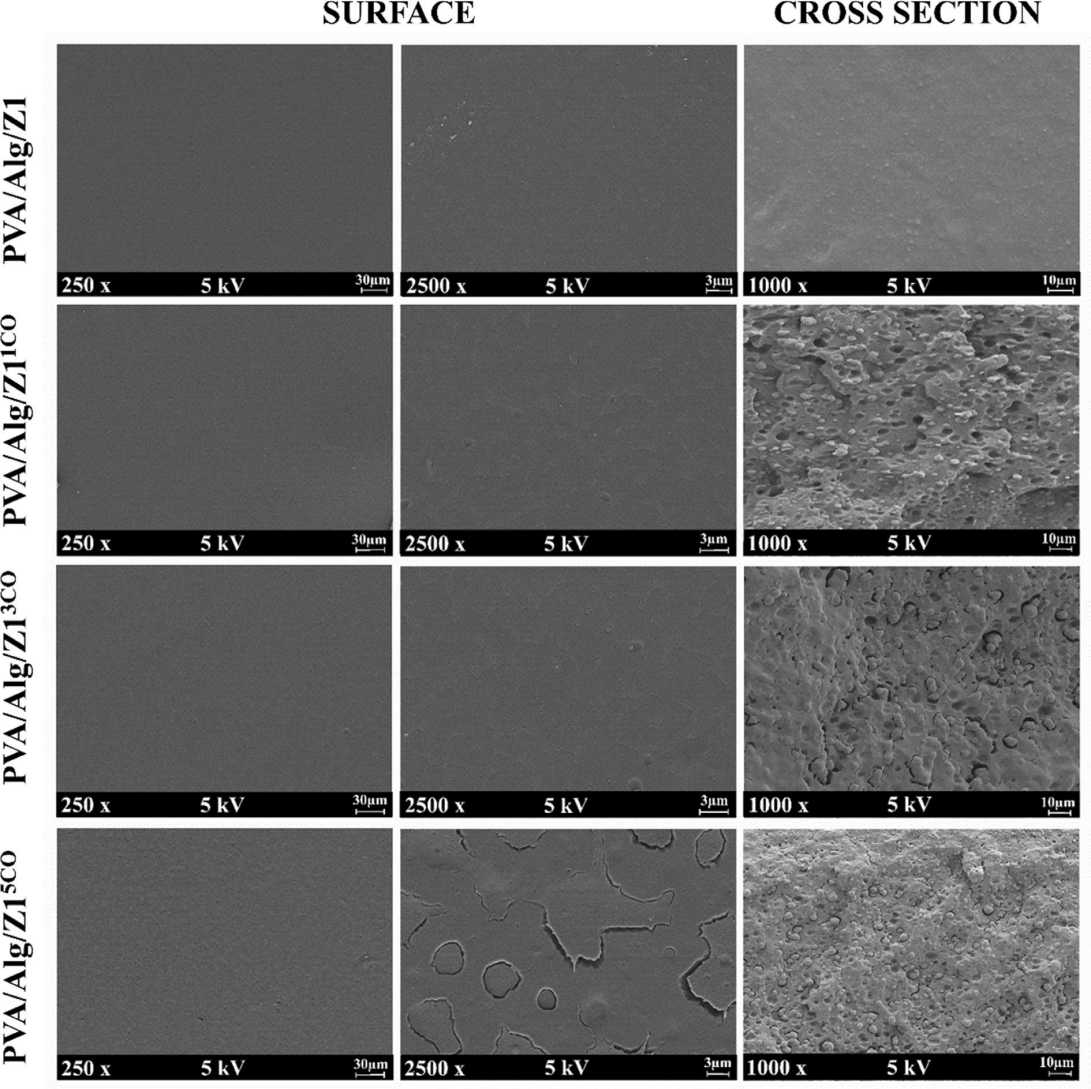
Surface and cross sectional morphology of the essential oil‐loaded Ag/ZnO‐PVA/Alg‐based hydrogels freezing at −18°C and thawing at 24°C.

The surface morphology of Ag/ZnO‐doped PVA/Alg‐based hydrogel wound dressings enriched with CO, prepared via the F–T process involving freezing at −18°C and thawing at 24°C, is presented in Figure 6. The SEM micrographs revealed a uniform and smooth surface morphology, suggesting good miscibility of the polymer components. Additionally, the essential oil, incorporated at varying concentrations—particularly at lower levels—in combination with Ag/ZnO, appeared to be uniformly distributed throughout the polymeric network, with no visible signs of phase separation or nanoparticle aggregation. The hydrogel containing 1 wt.% CO exhibited the most favorable results, as the oil droplets were effectively entrapped within the three‐dimensional network of the membrane. Given the inherently hydrophobic nature of essential oils and their immiscibility with water, an increase in CO concentration led to the appearance of visible, insoluble oil droplets. Notably, when the CO content exceeded 3%, the formation of macrovoids was observed. This phenomenon is attributed to the reduced efficiency of hydroxyl group interactions at higher concentrations and the increased influence of phenolic groups, which are less compatible with polar solvent environments [67].

Furthermore, the larger voids are likely associated with the removal or rearrangement of dispersed oil domains during sample drying prior to SEM observation, particularly at higher CO contents. These surface voids may increase the hydrogel's effective surface area, potentially enhancing oxygen permeability and facilitating improved gas exchange—an essential feature for wound healing. In addition, the porous architecture may support more efficient fluid exchange between the wound bed and the external environment, thereby helping to maintain moisture balance and prevent excessive exudate accumulation. Moreover, the presence of surface pores may enhance hydrogel adhesion to the wound bed, ensuring closer contact and potentially accelerating the healing process [5].

In addition to these oil‐related voids, the F–T process contributes to the formation of a porous architecture through ice‐crystal templating within the polymer matrix. The porous features observed in the SEM images are mainly attributed to F–T‐induced phase separation occurring in the PVA/water system [68]. During the freezing stage, water molecules crystallize and act as temporary porogens within the polymer matrix [69]. Upon thawing and subsequent drying, the removal of these ice crystals leaves voids that appear as pores in the hydrogel structure [70]. Although SEM observations are conducted on dried samples and partial structural shrinkage may occur, the interconnected porous morphology observed here is consistent with previously reported F–T PVA hydrogel systems. This process simultaneously generates physical crosslinking points through PVA microcrystallite formation while producing a porous architecture within the hydrogel network [71].

Additionally, cross‐sectional SEM images reveal that the PVA/Alg/Z1 sample has a dense and uniform structure without visible voids, indicating good compatibility between the polymer components and well‐dispersed ZnO particles. With the addition of CO, the internal morphology becomes progressively more porous. In PVA/Alg/Z1^1CO^ and PVA/Alg/Z1^3CO^, small cavities appear due to partial phase separation between the hydrophilic matrix and hydrophobic oil domains. At higher oil content PVA/Alg/Z1^5CO^, the structure shows larger voids and discontinuities, suggesting reduced matrix integrity but increased permeability. Overall, the incorporation of essential oil transforms the compact polymer structure into a more open and porous morphology, promising candidate for further evaluation as a wound dressing material.

Surface wettability was evaluated through contact angle measurements (CA) provides important insight into the surface characteristics of hydrogels and plays a critical role in their biological performance, particularly with respect to cell adhesion, proliferation, and wound healing efficacy. Hydrophilic surfaces with suitable wettability promote the gradual absorption of biological fluids exuding from the dermis when in contact with moist areas of a wound [72]. In general, a surface is considered hydrophilic if it exhibits a CA less than 90° [73].

**FIGURE 7:**
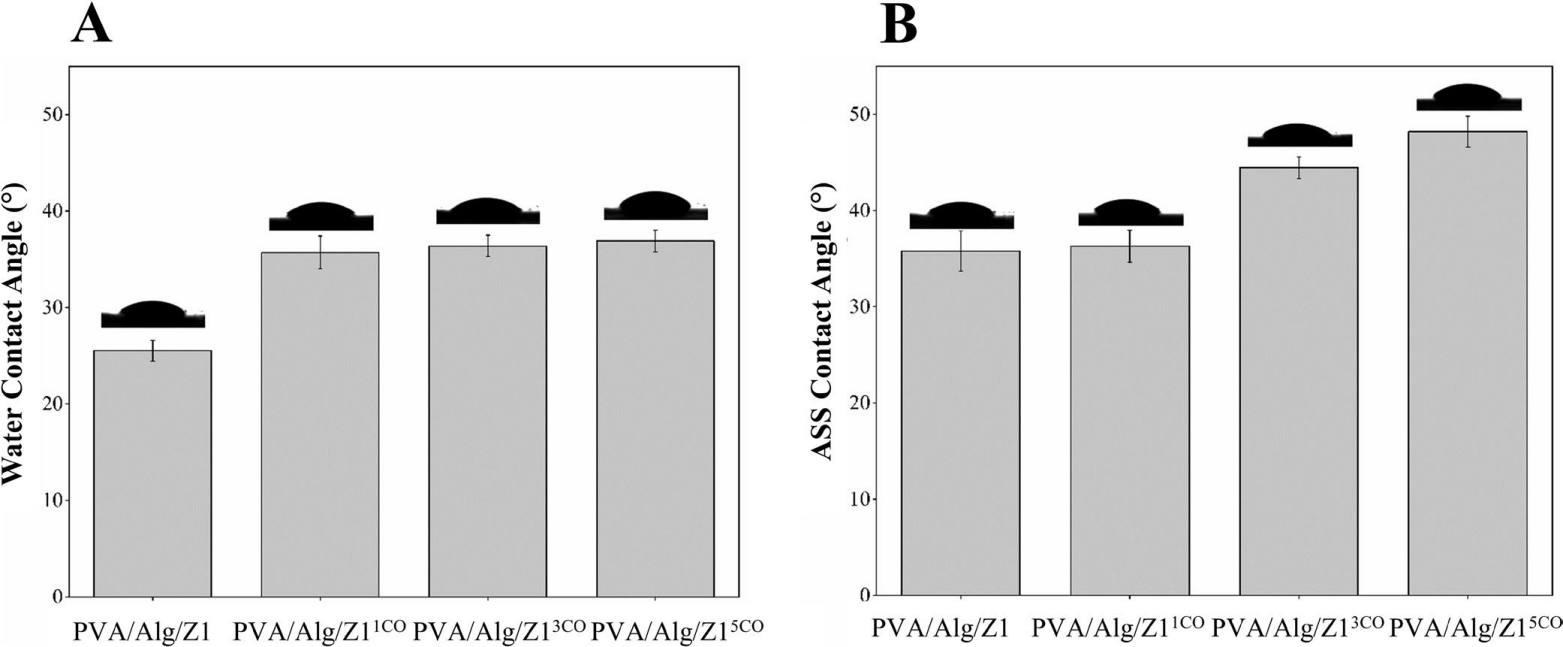
Surface properties of essential oil loaded Ag/ZnO‐PVA/Alg‐based hydrogels freezing at −18°C and thawing at 24°C: (A) Contact angle measurements in water, (B) Contact angle measurements in ASS.

The relative surface hydrophilicity of PVA/Alg‐based hydrogels was assessed via CA measurements using two different media—distilled water and artificial sweat solution—as presented in Figure 7A,B, respectively. Because hydrogel surfaces may absorb liquid droplets, the reported contact angle values represent the initial wettability of the surface. The PVA/Alg/Z1 hydrogel exhibited a water CA of 25.51° ± 1.1°, attributed to the inherently hydrophilic nature of the constituent polymers. Doping the essential oil into the polymer matrix led to a notable increase in water CA, reaching 35.7° ± 1.7° in the PVA/Alg/Z1^CO1^ sample. As the concentration of essential oil increased, the water CA further rose to 36.36° ± 1.12° and 36.9° ± 1.13° for the PVA/Alg/Z1^CO3^ and PVA/Alg/Z1^CO5^ samples, respectively. The progressive increase in water CA indicates a gradual decrease in surface hydrophilicity, which can be attributed to variations in the concentrations of the incorporated components across different hydrogel formulations as well as alterations in the surface roughness. The increase in CA can also be attributed to the inherently hydrophobic nature of the incorporated essential oil. Due to the hydrophobic nature of essential oil, an oil emulsion was prepared to improve its dispersion within the polymer matrix [74]. To provide a more comprehensive evaluation of hydrogel surface wettability, CA measurements were also performed in the presence of ASS. The lowest artificial sweat solution CA was observed for the neat PVA/Alg as 35.81° ± 2.1° whereas the highest was recorded for the PVA/Alg/Z1^CO5^ as 48.21° ± 1.6°. Nevertheless, the CA measurements below 90° confirmed that all hydrogel samples had hydrophilic surfaces in both water and artificial sweat media. This hydrophilicity is attributed to the polymer matrix consisting of PVA and Alg, which contains polar groups capable of absorbing water. Moreover, the slight decrease in wettability with increasing essential oil content may reduce excessive adhesion of hydrogel dressings to burn wounds, potentially facilitating less painful dressing removal [60]. On the other hand, the ability to alter the CA of hydrogel dressings by varying the concentration of additives makes it possible to use modified PVA/Alg‐based hydrogels for the delivery and release of biologically active agents [72].

#### 3.3.4 Structural and Thermal Properties of Essential Oil‐Ag/ZnO‐(PVA/Alg) Hydrogels

**FIGURE 8:**
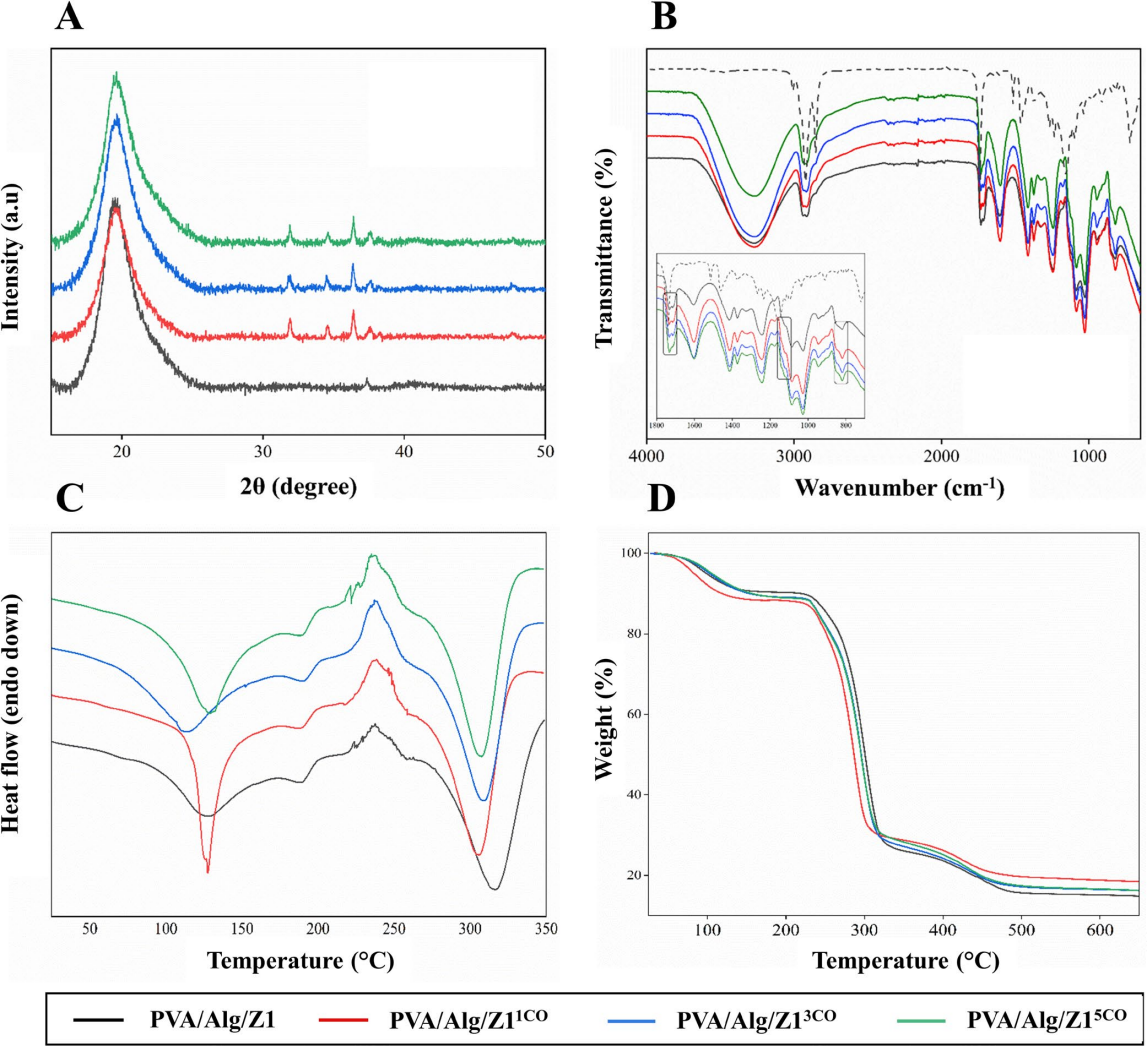
Structural and thermal properties of essential oil‐Ag/ZnO‐(PVA/Alg) hydrogels after freezing at −18°C and thawing at 24°C: (A) XRD patterns of the hydrogels, (B) FTIR spectra of the hydrogels, (C) DSC thermograms of the hydrogels, and (D) TGA thermograms of the hydrogels.

XRD analysis provides insight into the structural characteristics of hydrogels, including their crystallinity and phase composition [75]. Figure 8A presents the XRD patterns of the synthesized hydrogels. The neat PVA/Alg hydrogel exhibits a broad diffraction peak in the range of 18°–22°, which is indicative of the amorphous nature of both PVA and Alg. The absence of sharp diffraction peaks in the neat sample suggests a disordered arrangement of polymer chains [75, 76].

In contrast, hydrogels doped with Ag/ZnO powders displayed distinct crystalline peaks at about 2*θ* = 31.75°, 34.41°, 36.23°, and 47.52°, which are absent in the neat sample. These peaks confirm the successful incorporation of Ag/ZnO powders into the polymer matrix [75]. Additionally, slight shifts in peak position and changes in intensity were observed, indicating possible lattice distortions resulting from nanoparticle incorporation. The XRD patterns of the Ag/ZnO‐doped PVA/Alg hydrogels further suggest improved dispersion of the nanoparticles within the physically cross‐linked hydrogel network. Notably, the XRD patterns showed no distinct peaks corresponding to essential oils, confirming their non‐crystalline structure. These results indşcate that variations in essential oil concentration do not significantly alter the lamellar organization within the polymeric network [57].

FTIR spectrum serves as a molecular fingerprint of a material, revealing the vibrational modes associated with its chemical constituents. Each peak corresponds to a specific functional group, characterized by its unique infrared absorption pattern [77, 78]. Figure 8B shows the FTIR spectra of the hydrogel dressings, while Table 3 summarizes the corresponding peak assignments.

**TABLE 3:** Characteristic FTIR peak assignments of essential oil‐Ag/ZnO‐(PVA/Alg) hydrogels.

Wavenumber (cm^−1^)		Structural units	Ref.
PVA/Alg/Z1	PVA/Alg/Z1^1CO^		
	PVA/Alg/Z1^3CO^		
	PVA/Alg/Z1^5CO^		
3100–3600	3100–3600	O—H stretching vibrations (PVA, eugenol)	[79, 80]
2941	2941	CH_2_ asymetric and symmetric vibrations (PVA)	[79]
2930	2930	CH_2_ vibrations (Alg)	[79]
—	2920	CF_2_ streching vibrations (eugenol)	[80]
	1765	Ester group (eugenol acetate)	[80]
—	1637	CF_2_ streching vibrations (eugenol)	[80]
1613	1613	COO^−^ asymmetric stretching (Alg)	[79]
1608	1608	Zn—OH (Ag/ZnO)	[81]
1416	1416	COO^−^ symmetric stretching (Alg)	[79]
—	1269	C—O stretching (eugenol)	[80]
1093	1093	C—O stretching (PVA)	[79]
1037	1037	C—O stretching (Alg)	[79]
750–400	750–400	Zn—O stretching (Ag/ZnO)	[81]

The FTIR spectra of PVA/Alg‐based hydrogels display characteristic absorption features associated with both polymeric constituents. A broad band extending from approximately 3100 to 3600 cm^−1^ originates from O—H stretching vibrations of hydroxyl groups along the PVA backbone, consistent with hydrogen bonding interactions within the hydrogel network. Bands detected near 2940–2930 cm^−1^ are attributed to C—H stretching vibrations arising from both PVA and Alg macromolecular chains. Distinct absorption peaks observed at around 1437 and 1093 cm^−1^ are assigned to CH—OH bending and C—O stretching modes, respectively, while signals at approximately 1613 and 1416 cm^−1^ are assigned to the asymmetric and symmetric stretching vibrations of Alg carboxylate (—COO^−^) groups. An additional band near 1037 cm^−1^ further confirms C—O stretching contributions from Alg. These spectral signatures are in good agreement with previously reported FTIR characteristics of PVA/Alg hydrogel systems [79].

Upon incorporation of Ag/ZnO and CO, the FTIR spectra retain the main polymer‐related absorption bands, indicating that the fundamental chemical structure of the PVA/Alg network remains intact. In Ag/ZnO‐containing hydrogels, an absorption band around 1608 cm^−1^ is commonly attributed to Zn—OH related interactions. Bands in the 400–750 cm^−1^ region are associated with Zn—O lattice vibrations, confirming the presence of the metal oxide phase within the hydrogel matrix [72]. In addition, characteristic absorption features related to eugenol, the major bioactive constituent of clove oil, are detected, supporting the successful incorporation of the essential oil without chemical degradation [81]. Furthermore, the characteristic peaks of eugenol which is the primary component of CO, were clearly observed in the Ag/ZnO‐doped PVA/Alg‐based hydrogels enriched with CO. The bands at approximately 3432, 3071, and 2920 cm^−1^ were assigned to O—H, =C—H, and C—H stretching vibrations, respectively. Peaks at 1637 and 1606 cm^−1^ corresponded to the C=C stretching vibrations of the aromatic ring. The band at 1431 cm^−1^ was attributed to CH_2_, while the one at 1269 cm^−1^ was related to C—O stretching. The peaks at 990 and 804 cm^−1^ arose from CH out‐of‐plane and ring deformations. Additionally, the bands at 1637 and 995 cm^−1^ were associated with the stretching vibrations of the allyl group. A peak observed at 1765 cm^−1^ was assigned to the ester group of eugenol acetate, while the band between 1100 and 1210 cm^−1^ was attributed to asymmetric C—O—C stretching of eugenol [80]. These spectral features suggest interfacial interactions between Ag/ZnO particles and the hydroxyl/carboxyl groups of the PVA/Alg network, which may contribute to restricted chain mobility and improved structural integrity of the composite hydrogel. CO can interact with the hydrogel network through both physical entrapment and intermolecular interactions. The phenolic hydroxyl group of eugenol may form hydrogen bonds with hydroxyl groups of PVA and carboxylate groups of Alg [82]. In addition, the hydrophobic aromatic structure of eugenol allows partial physical entrapment of CO droplets within the pores of the hydrogel network formed during F–T processing [43]. These interactions indicate that CO retention in the hydrogel matrix is governed by a combination of hydrogen bonding and physical entrapment mechanisms.

During F–T processing, the crosslinking of PVA‐based hydrogels occurs through the formation of PVA microcrystallites that act as physical junction points within the polymer network [83, 84]. The crystallization of PVA chains during the freezing stage leads to phase separation and the formation of ordered crystalline domains that stabilize the three‐dimensional network upon thawing. Alg chains contribute to network stabilization mainly through hydrogen bonding interactions with the hydroxyl groups of PVA as well as through physical chain entanglements [39]. The incorporation of Ag/ZnO particles can further influence the network structure through interfacial interactions between the filler surface and the polymer chains. In particular, surface hydroxyl groups on ZnO may interact with the hydroxyl groups of PVA and the carboxylate groups of Alg through hydrogen bonding or coordination interactions [85]. These filler–polymer interactions restrict chain mobility and promote a more compact network structure, which can be interpreted as an increase in the effective crosslink density of the hydrogel. These findings confirm the successful incorporation of Ag/ZnO and CO into the PVA/Alg hydrogel matrix and support the presence of intermolecular interactions contributing to the stability of the composite network.

Differential scanning calorimetry (DSC) is a powerful analytical technique for evaluating the thermal behavior of hydrogel‐based wound dressings, providing information on phase transitions and polymer–additive interactions. Accordingly, DSC analysis was applied to investigate the thermal response of the prepared hydrogel formulations. The DSC heating profiles are presented in Figure 8C, and the extracted thermal parameters, including glass transition temperature (Tg), melting temperature (Tm), and melting enthalpy (ΔHm), are listed in Table 2. The PVA/Alg/Z1 hydrogel displayed a glass transition temperature of 65.33°C and a melting temperature of 126.67°C, with a corresponding fusion enthalpy (ΔHm) of 100.26 J g^−1^. Incorporation of clove oil led to a gradual decrease in Tg values to 57.68°C, 52.40°C, and 52.01°C for hydrogels containing 1, 3, and 5 wt.% CO, respectively. This reduction indicates a plasticizing effect that increases the mobility of polymer chains. The Tm values for the PVA/Alg/Z1, PVA/Alg/Z1^1CO^, and PVA/Alg/Z1^5CO^ samples were in the range of 126°C–128°C. In contrast, the PVA/Alg/Z1^3CO^ sample showed a lower Tm of 113.31°C, indicating reduced thermal stability of its crystalline domains. ΔHm values were higher in all CO‐containing samples compared to the PVA/Alg/Z1 sample. Notably, the highest value, 179.42 J/g, was observed for the PVA/Alg/Z1^5CO^ sample, suggesting that CO may promote the formation or perfection of crystalline regions in the PVA matrix. Overall, CO addition decreased Tg, increased ΔHm, and induced composition‐dependent variations in Tm, consistent with observations for other essential oil–plasticized biopolymer systems [86].

Figure 8D shows the TGA profiles exhibited three characteristic stages of weight loss: moisture evaporation (approximately 50°C–150°C), polymer chain decomposition (around 200°C–350°C), and carbonization at higher temperatures (above 400°C). Compared to the neat PVA/Alg, the CO‐loaded samples displayed slightly lower onset degradation temperatures, likely due to the disruption of intermolecular hydrogen bonding and the introduction of thermally labile organic constituents by essential oil. Notably, the residual mass increased with rising CO content, which can be attributed to the aromatic structure of eugenol and other phenolic compounds in clove oil that facilitate char formation during thermal degradation. Overall, the thermal stability followed the order PVA/Alg > 1CO > 3CO > 5CO, indicating that higher CO incorporation enhances charring efficiency while slightly reducing the thermal stability of the polymer matrix. These thermal characteristics may also be associated with changes in the internal structure of the hydrogel network. The plasticizing effect of CO increases polymer chain mobility, while the formation of hydrophobic domains within the matrix may partially restrict water vapor diffusion and influence oxygen transport behavior. These structural modifications are consistent with the reduced WVTR and composition‐dependent OTR values observed in the barrier characterization.

#### 3.3.5 Barrier Properties of Essential Oil‐Ag/ZnO‐(PVA/Alg) Hydrogels

In wound environments, fluid loss is primarily governed by diffusion from sweat glands and areas with low relative humidity. Specifically, in burn wounds, the loss of the epidermal barrier significantly increases transepidermal water loss—the passive evaporation of water vapor from the skin surface—thereby impairing the healing process. Hydrogel dressings help mitigate fluid loss by maintaining a moist environment beneath the wound and promoting epithelialization. For wound dressings designed specifically for burn injuries, barrier properties such as water vapor and oxygen permeability are critical parameters that directly influence the healing process. An ideal wound dressing should prevent dehydration of the wound bed while enabling the controlled evaporation of excess exudate, and at the same time ensure adequate oxygen delivery to the wound site to support cell proliferation and angiogenesis [74].

Table 4 presents the WVTR, WVP, and OTR results of Ag/ZnO‐doped PVA/Alg‐based hydrogels loaded with CO. The loading of CO into the hydrogel formulation led to a decrease in WVTR. For instance, while the neat Ag/ZnO‐doped PVA/Alg‐based sample (PVA/Alg/Z1) exhibited a WVTR of 601.39 g·m^−2^·day^−1^, this value decreased to 383.42 g·m^−2^·day^−1^ in the sample containing 5% CO (PVA/Alg/Z1^5CO^). Similarly, the WVP—calculated based on sample thickness and water vapor pressure—decreased from 60.54 g·mm/m^2^·day·kPa in the PVA/Alg/Z1 sample to 38.6 g·mm/m^2^·day·kPa in the PVA/Alg/Z1^5CO^ sample. This reduction can be attributed to the hydrophobic nature of CO, which likely causes partial pore blockage within the hydrogel matrix restricting water vapor diffusion. This effect became more pronounced at higher CO concentrations. Similarly, previous studies reported that wound dressings containing PVA and polyvinyl pyrrolidone (PVA/PVP) had a WVTR of approximately 625 g/m^2^·day, while when different essential oils were incorporated into the polymer matrix, the WVTR of the sample containing the highest oil content was measured as approximately 135 g/m^2^·day. This decrease suggests that the incorporation of essential oil creates a barrier effect [48]. Given that the WVTR values suitable for skin and wound types range between 76 and 9360 g·m^−2^·day^−1^, and that wounds with moderate exudate typically require a WVTR between 904 and 1447 g·m^−2^ day^−1^. These values fall within the broad WVTR range reported for wound dressings (76–9360 g·m^−2^·day^−1^) and suggest that the developed hydrogels may be suitable particularly for wounds with low to moderate exudate levels, where excessive dehydration of the wound bed should be avoided [87].

**TABLE 4:** Water vapor transmission rate (WVTR), water vapor permeability (WVP), and oxygen transmission rate (OTR) of essential oil‐Ag/ZnO‐(PVA/Alg) hydrogels.

Sample code	Thickness (mm)	WVTR (g·m^−2^·day^−1^)	WVP (g·mm/m^2^·day·kPa)	OTR (cm^3^·mm·m^−2^·day^−1^·kPa^−1^)
PVA/Alg/Z1	0.199 ± 0.004	601.39	60.54	23.22
PVA/Alg/Z1^1CO^	0.205 ± 0.001	446.18	44.93	64.25
PVA/Alg/Z1^3CO^	0.203 ± 0.009	407.87	41.05	56.11
PVA/Alg/Z1^5CO^	0.210 ± 0.004	383.42	38.6	44.27

Additionally, the Ag/ZnO‐loaded PVA/Alg‐based sample containing 1 wt.% CO exhibited an optimal balance in terms of both OTR (64.25 cm^3^·mm·m^−2^·day^−1^·kPa^−1^) and WVTR (446.18 g/m^2^·day). At this concentration, the essential oil is likely to have formed micro‐pores within the polymer matrix, enhancing oxygen diffusion while maintaining sufficient vapor permeability to prevent excessive moisture loss. In contrast, higher CO concentrations led to reductions in WVTR, WVP, and OTR values, suggesting that excessive oil content may induce phase separation and morphological disruption in the sample structure. Similar findings have been reported in the literature, where essential oils at appropriate concentrations provided wound‐healing and antimicrobial effects, while also playing a critical role in maintaining moisture and oxygen balance at the wound site [88, 89]. Sample thickness strongly influences transmission measurements; therefore, WVTR results were interpreted together with WVP and oxygen transport parameters to allow more reliable comparisons between formulations.

Taken together these findings indicate that CO loading modulates the barrier performance of Ag/ZnO–(PVA/Alg) hydrogels through hydrophobic and plasticizing effects. The sample containing 1 wt.% CO achieved the most favorable combination of WVTR and OTR, offering balanced water retention and oxygen diffusion—an essential requirement for effective burn wound healing. Compared with the neat PVA/Alg matrix (PVA/Alg/Z1), the CO‐containing formulations exhibited tunable reductions in WVTR and increased oxygen transmission, suggesting that the composite design enables simultaneous regulation of barrier and transport properties. Notably OTR measurements were conducted at 0% relative humidity to ensure instrument stability and to provide a consistent comparative screening of the formulations. However, hydrated or high‐humidity conditions may better represent the physiological wound environment. This limitation is acknowledged, and future studies will investigate oxygen transport behavior under hydrated conditions. In the present study, sample thickness and thickness‐normalized transport parameters were reported to enable more reliable comparisons with literature and clinical benchmarks. The primary purpose of these measurements was to enable comparative evaluation of the different formulations under identical experimental conditions. These results highlight the potential of essential oil–loaded Ag/ZnO–(PVA/Alg) hydrogels as tunable wound dressing materials with balanced moisture management and oxygen transport properties.

#### 3.3.6 In Vitro Biological Properties Essential Oil‐Ag/ZnO‐(PVA/Alg) Hydrogels

Effective management of wound exudate—the fluid released during the healing process—is essential for successful wound care. Excess exudate accumulation can impair microvascular perfusion and elevate protease levels, which degrade the extracellular matrix (ECM), leading to tissue damage. Furthermore, the moist and nutrient‐rich environment created by excess exudate promotes microbial (bacterial and fungal) growth, inflammation, and delays healing, potentially resulting in non‐healing wounds [5]. In this context, the capacity of wound dressings to absorb exudate is a critical determinant for accelerating wound healing. To evaluate this property, the CO loaded Ag/ZnO‐doped PVA/Alg‐based hydrogel dressings were tested for their fluid uptake using SWF, with swelling percentage calculations serving as an indirect measure of exudate absorption ability.

**FIGURE 9:**
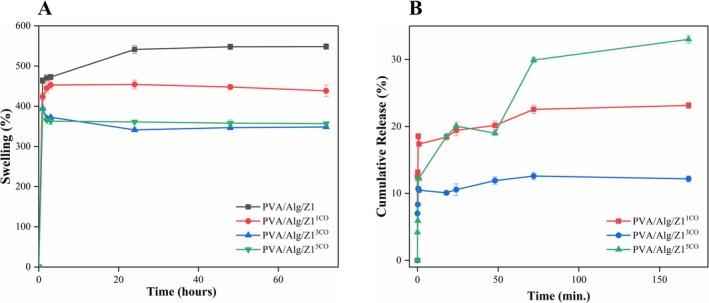
In vitro biological properties of essential oil‐Ag/ZnO‐(PVA/Alg) hydrogels after freezing at −18°C and thawing at 24°C: (A) Swelling degree of the hydrogels at different time intervals, (B) Cumulative release of the hydrogels at different time intervals.

As shown in Figure 9A, PVA/Alg exhibits the highest swelling degree, which can be attributed to the hydrophilic nature of both PVA and Alg, as explained in CA results. When essential oil was incorporated into the hydrogels, the swelling degree decreased compared to the neat PVA/Alg sample due to the presence of hydrophobic CO domains. Nonetheless, the swelling levels observed across all hydrogel formulations remained adequate for wound dressing application. The swelling profiles indicated a rapid uptake during the first few hours, reaching equilibrium around 24 h, followed by a gradual stabilization. After 48 and 72 h of immersion, only slight variations in swelling percentage were recorded, suggesting that the hydrogel network attained a dynamic equilibrium where water absorption and relaxation forces became balanced. This plateau behavior demonstrates the structural stability of the crosslinked matrix and its ability to maintain moisture over prolonged exposure without disintegration—an important criterion for burn wound applications requiring sustained hydration. Furthermore, the swelling degree of the hydrogels diminished gradually after 24 h. This behavior likely results from increased internal pressure in the polymeric network, causing water to be expelled from weaker regions as chain dissociation occurs, ultimately reducing swelling over time [5]. These observations are consistent with findings from other systems, such as thymol‐loaded chitosan/gelatin and *Zataria multiflora* essential oil–loaded PVA/gelatin hydrogels, which similarly demonstrate declining swelling behavior over time due to polymer network relaxation and hydrophobic additive effects [90, 91].

The assessment of essential oil release from the polymeric carrier requires prior quantitative analysis of the prepared CO emulsion to accurately establish the incorporated oil content in hydrogels with different loadings. The measured concentrations, which directly correlated with the initial CO content of the samples, were used as reference values for calculating the cumulative release profiles.

The amount of released CO was quantified by UV–visible spectroscopy at 280 nm, corresponding to the characteristic absorption of eugenol, the major phenolic component of CO. A calibration curve was established using CO emulsions of known concentrations prepared under identical experimental conditions. To minimize possible spectral interference, blank measurements containing PBS and Tween 80 without CO were recorded under identical conditions and used for baseline correction. In addition, control hydrogels without CO were analyzed to verify that PVA/Alg components did not contribute significantly to the absorbance at 280 nm. These controls confirmed that the monitored signal primarily originated from released CO.

The release behavior of CO from the hydrogel matrices was evaluated using a total immersion method in PBS. The release profiles were monitored and expressed as cumulative oil release over time.

Representative release curves, together with visual changes in hydrogel morphology as a function of immersion duration, are presented in Figure 9B. The release profiles exhibited a characteristic biphasic pattern consisting of an initial burst phase followed by a slower and more controlled stage. During the first minute, hydrogels containing 1%, 3%, and 5% CO released approximately 12%, 7%, and 4% of the loaded oil, respectively, indicating a concentration‐dependent burst effect. This rapid initial release is primarily attributed to the diffusion of CO molecules located near the hydrogel surface, together with the rapid water uptake and swelling of the hydrophilic PVA/Alg network, which facilitates early desorption of loosely bound oil molecules [92]. Following the burst phase, the release rate decreased markedly and approached a plateau region, indicating a transition to diffusion‐controlled transport from the inner polymer matrix. This behavior suggests that the remaining oil was predominantly entrapped within the inner polymer matrix and released via a slower diffusion‐controlled mechanism. Consistent with this interpretation, the kinetic parameters summarized in Table 5 further support the biphasic release behavior of the developed hydrogels. Two‐phase kinetic analysis showed that the PVA/Alg/Z1^5CO^ sample exhibited excellent agreement with the Higuchi model in Phase II (*R* ^2^ ≈0.999), while the Korsmeyer–Peppas exponent obtained for the early stage (*n* = 0.128) confirmed predominantly Fickian diffusion‐controlled release [93, 94].

**TABLE 5:** Two‐phase release kinetic parameters of essential oil‐Ag/ZnO‐(PVA/Alg) hydrogels based on Higuchi and Korsmeyer–Peppas models.^a^

Sample code	Phase	Higuchi kH	Higuchi *R* ^2^	KP (*k*, *n*, *R* ^2^)	Mechanism
PVA/Alg/Z1^1CO^	Phase I (1–30 min)	9.389	0.692	—	Burst‐dominated
PVA/Alg/Z1^3CO^	Phase I (1–30 min)	6.054	0.900	—	Burst‐dominated
PVA/Alg/Z1^5CO^	Phase I (1–30 min)	12.818	0.787	*k* = 0.352, *n* = 0.128, *R* ^2^ = 1.000	Fickian diffusion
PVA/Alg/Z1^1CO^	Phase II (1–24 h)	0.460	0.887	—	Slow diffusion/Plateau behavior
PVA/Alg/Z1^3CO^	Phase II (1–24 h)	−0.024	0.037	—	Plateau behavior
PVA/Alg/Z1^5CO^	Phase II (1–24 h)	1.974	0.999	—	Controlled diffusion

^a^ Higuchi model was fitted as Mt = k_H_t^1/2^ Korsmeyer–Peppas fitting was performed only within its validity range (Mt/M∞ ≤ 0.6M); therefore, it was applicable only to Z15CO in Phase I due to the pronounced burst release in Z11CO and Z13CO. Time was expressed in hours.

The hydrophilic nature of PVAand Alg further supports to the initial burst release by facilitating water uptake and polymer swelling. After the early burst, the release profile reached a plateau and remained relatively constant across all CO‐loaded formulations, indicating a sustained release behavior beneficial for prolonged therapeutic activity. Among the formulations, PVA/Alg/Z1^3CO^ reached an early plateau with minimal variation over time, limiting the applicability of classical kinetic models in the later stage. Accordingly, the slightly negative Higuchi slope calculated for PVA/Alg/Z1^3CO^ in Phase II is most plausibly attributed to the near‐steady‐state release condition and the reduced concentration gradient, rather than to a true reverse diffusion phenomenon.

In addition to the plateau‐dominated profiles observed in some formulations, the release behavior is further influenced by the permeability of the polymer matrix and the shortened diffusion pathways provided by the porous structure. For example, the PVA/Alg/Z1^5CO^ sample exhibited relatively low initial release followed by a marked increase after 18 h. This behavior is more plausibly associated with the gradual evolution of diffusion pathways within the swollen hydrogel network rather than simple pore blockage, as the presence of large surface macropores can progressively facilitate outward oil diffusion over time.

Collectively, the developed hydrogels demonstrate a burst release governed by surface‐associated oil, followed by diffusion‐limited transport through the swollen polymer network. This release profile is advantageous for achieving sustained therapeutic activity in wound dressing applications. When considered together with the swelling and barrier results, the present findings indicate that the incorporation of CO and Ag/ZnO enables concurrent modulation of moisture management, oxygen transport, and controlled release behavior. This integrated response supports the multifunctional potential of the composite hydrogel system relative to single‐component matrices.

From a translational perspective, further investigations are required to evaluate the environmental stability of CO within the hydrogel matrix, particularly with respect to volatility and oxidative degradation during storage. In addition, long‐term shelf stability, sterilization compatibility, and controlled Ag ion release behavior will need to be systematically examined to support future clinical translation of the developed hydrogel system.

## 4 Conclusions

This study demonstrates that Ag/ZnO–(PVA/Alg)‐based hydrogels enriched with essential oil can be effectively fabricated via a solvent‐free F–T process, yielding physically cross‐linked networks with favorable mechanical performance and structural stability. The incorporation of Ag/ZnO significantly enhanced gel fraction and tensile strength, suggesting its reinforcing effect on the polymer network through strong polymer–particle interactions. Clove oil incorporation governed barrier behavior, resulting in reduced water‐vapor permeability and an optimal oxygen transmission rate at 1 wt.% CO, indicative of a tunable mass‐transport balance. Swelling experiments revealed rapid fluid uptake and sustained hydration stability, while CO release studies indicated sustained release behavior over time. Thermal and structural analyses confirmed the successful integration of Ag/ZnO and CO without compromising polymer chain stability. The results establish clear composition–structure–property relationships in Ag/ZnO‐containing PVA/Alg hydrogels and highlight the promise of F–T processed hybrid systems with adjustable physicochemical and transport properties for further investigation in wound dressing‐related applications.

## Funding

This research received no external funding.

## Conflicts of Interest

The author declares no conflicts of interest.

## Data Availability

The data that support the findings of this study are available from the corresponding author upon reasonable request.
